# A case of childhood hyperekplexia due to a novel nonsense variant in the GLRA1 gene

**DOI:** 10.1097/MD.0000000000046940

**Published:** 2026-01-23

**Authors:** Shuang-Zhu Lin, Xiao-Yu Sun, Yang-Yang Tan, Yang-Fan Qi, Kai Jiang

**Affiliations:** a College of Traditional Chinese Medicine, Changchun University of Chinese Medicine, Changchun, Jilin Province, China.

**Keywords:** case report, children, epilepsy, *GLRA1*, hyperekplexia

## Abstract

**Rationale:**

Hyperekplexia is a rare hereditary neurological disorder characterized by an exaggerated startle response and generalized rigidity. It is frequently misdiagnosed as epilepsy, leading to unnecessary treatment. We report a case of mild hyperekplexia caused by a novel *GLRA1* mutation, highlighting the diagnostic value of genetic testing in atypical presentations.

**Patient concerns:**

An 18-month-old girl presented with recurrent episodes of vacant staring and limb rigidity triggered by sudden sounds or tactile stimuli over the past 2 months. She had a history of 3 febrile seizures but normal development. Physical examination revealed hypersensitivity to external stimuli, but the nose-tap test was negative.

**Diagnoses:**

Routine metabolic studies, brain magnetic resonance imaging, and electroencephalogram were normal. Whole-exome sequencing identified a de novo heterozygous nonsense variant c.593G > A (p.Trp198Ter) in the *GLRA1* gene, confirming the diagnosis of hyperekplexia.

**Interventions:**

Given the confirmed diagnosis and the mild nature of the symptoms, the previously prescribed antiepileptic drug (levetiracetam) was discontinued. Clonazepam was not initiated due to the mild severity of the condition.

**Outcomes:**

At the 8-month follow-up, the patient remained seizure-free with minimal non-epileptic stimulus-induced symptoms. Her growth and psychomotor development were normal.

**Lessons:**

This case expands the phenotypic spectrum of *GLRA1*-related hyperekplexia to include mild phenotypes with a negative nose-tap test. It underscores the critical role of whole-exome sequencing in distinguishing hyperekplexia from epilepsy, thereby avoiding inappropriate antiepileptic therapy.

## 1. Introduction

Hyperekplexia is a rare autosomal hereditary neurological disorder, which belongs to the heterogeneous disorders of abnormal and excessive response to sudden external stimuli, such as stimulus-induced disorders and neuropsychiatric shock syndromes, etc. Its main clinical symptoms include muscle rigidity, startle reflex, apnea, convulsions and blinking.^[1]^ The main clinical symptoms include muscle rigidity, startle reflex, apnea, convulsions and episodic blinking. So far, studies have shown that *GLRA1*, *SLC6A*, *GPHN* and *GLRB* are the 4 causative genes involved.^[2]^ Among them, *GLRA1* is the main causative gene of the disease.^[3]^ Early diagnosis is very important for the treatment and prognosis of this disease.

## 2. Case presentation

Chief complaint: An 18-month-old girl presenting with 3 episodes of recurrent convulsions accompanied by vacant staring over the past 2 months.

History: The patient’s history began at 16 months of age when she experienced a convulsive seizure 1 day after the onset of fever. The episode was characterized by loss-of-consciousness, staring, trismus, and head tilting, lasting approximately 15 minutes and resolving spontaneously, accompanied by urinary and fecal incontinence. Two similar febrile-associated convulsive episodes recurred at approximately 17 months and 17.5 months of age, each occurring within 1 day of fever onset and resembling the initial event in both presentation and duration. She was previously diagnosed with “febrile seizures, suspected encephalitis” at a local hospital. After symptomatic treatment, her condition improved, and she was discharged with a prescription for levetiracetam. Currently, the child is alert with clear consciousness. Her hearing and vision are normal, though she occasionally exhibits vacant staring. Her appetite is slightly decreased, but bowel and bladder functions remain normal.

Past history: the child was in good health, 1st birth, 1st term, delivered by cesarean section, BW 3.2kg, BH 50cm.

Personal and family history: The child’s parents are healthy. The mother had an uneventful pregnancy and a natural delivery, without the use of any special medications or exposure to known toxic agents. The family history is unremarkable for similar disorders or other hereditary diseases. Her growth and developmental milestones were comparable to those of her peers.

Physical examination: H:81cm(25th-50th percentile), W:11kg (50th-75th percentile), and head circumference 47 cm (50th percentile). Clear, mentally available, good response, no special facial features, stable respiration, bilateral pupils equal in size and round, 3.0 mm in diameter, sensitive to light reflex, no nystagmus, normal eyelids. Skin elasticity was good all over the body, no hyperkeratosis, no hyperpigmentation of the skin and mucous membranes, no milky coffee spots, and no cumbersome skin on the hands and feet. Heart sounds were strong, heart rate 112 beats per minute, rhythmic, no additional heart sounds were heard, no pathological murmurs were heard. The rest of the lungs, abdomen, and anterior chest showed no obvious abnormalities. In gross motor, the child could walk alone, and in fine motor, he could build 2 to 3 blocks. Intellectually and linguistically, the child could carry out simple instructions and speak single words. The muscle strength and tone of the limbs were normal, physiological reflexes were present, and pathological reflexes were absent.

Physicochemical examination: (2024.6.25) Blood routine, urine routine, methyl function, liver and kidney function cardiac enzymes, blood homocysteine, blood ammonia, blood lactate, blood and urine genetic metabolism, head and hippocampal magnetic resonance imaging did not show any abnormality. (2024.7.3) Electroencephalogram showed predominantly bilateral anterior cephalic slow wave issuance during wakefulness, and bilateral frontal and frontal midline atypical spike wave and spike slow wave issuance during sleep. Head magnetic resonance imaging showed no abnormalities.

Further diagnostic examination: During physical examination, we found that the child was overly sensitive to external sounds and tactile stimuli, with occasional triggering of dull eyes and stiff limbs, and a negative nose-pointing test. When asked about his medical history, the family reported that the child was easily awakened at night during infancy and was sensitive to sound, but there was no history of choking and asphyxia, and there was no inguinal hernia or umbilical hernia.

The child has had 3 recurrent seizures in the past. Each seizure occurred within 24 hours of the onset of fever, strongly suggesting a high possibility of febrile seizures. However, the vacant stare episodes were not consistent with the EEG findings.

The physical examination revealed hypersensitivity to external stimuli, and although the child’s nasal puncture test was negative, it led us to think about the possibility of excessive startle response syndrome.

After obtaining parental consent, peripheral blood samples were collected from the proband and her parents, from which genomic DNA was extracted for whole-exome sequencing. Whole-exome sequencing was performed using the Illumina NovaSeq 6000 platform, achieving a mean coverage depth of > 100x across the exonic regions. Bioinformatic analysis included sequence alignment with the BWA and GATK. A stepwise filtering strategy was applied to prioritize candidate variants: we excluded common polymorphisms with an allele frequency > 0.1% in the gnomAD database. The identification and interpretation of the GLRA1 variant were strictly conducted in accordance with the standards and guidelines of ACMG/AMP. The variant was subsequently confirmed by Sanger sequencing, which was also used to evaluate its segregation pattern within the family.

**Figure 1.**
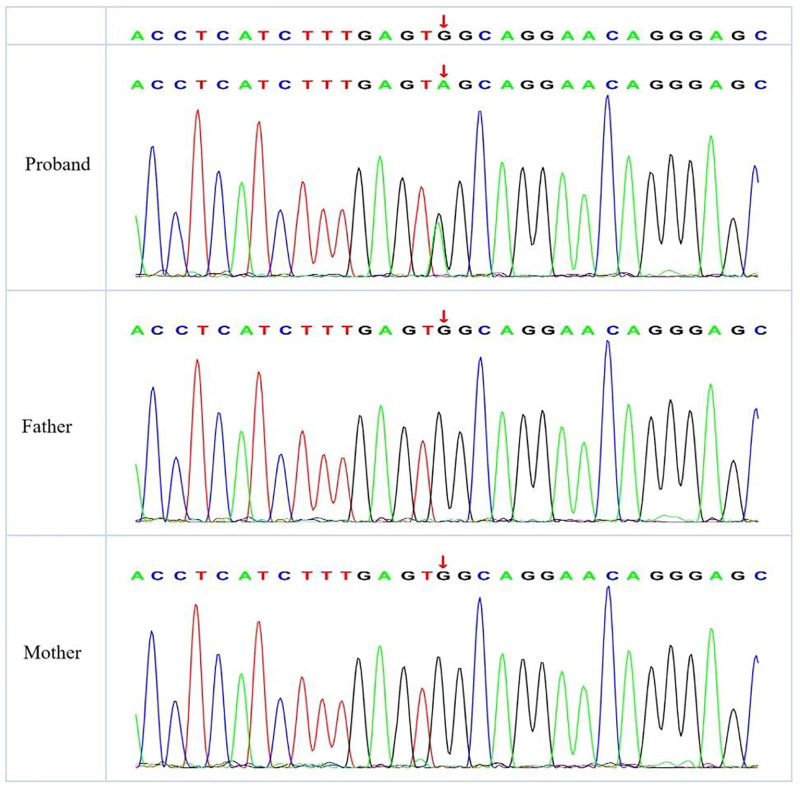
Sanger sequencing results for the pathogenic variant gene of the proband and his parents

The whole-exon testing suggested a new heterozygous variant in the *GLRA1* gene, in which the child’s parents were wild-type, c..593 (exon6)G > A [p.(Trp198Ter,252)], which replaced the nucleotide at position 593 of the coding sequence of the *GLRA1* gene from guanine (G) to adenine (A), resulting in a truncating mutation in the protein (Fig. 1). The amino acid at position 198 was changed from a tryptophan (Trp) to a termination codon (Ter), a truncating mutation. This variant was classified as pathogenic according to the ACMG/AMP guidelines, based on the following evidence: PVS1 (null variant [nonsense] in a gene where loss-of-function is a known mechanism of disease), PS2 (de novo occurrence in a patient with the disease and no family history), and PM2 (absent from population databases such as gnomAD).

Final diagnosis: hyperekplexia.

Treatment: Considering the patient’s clinical manifestations, we diagnosed the patient with hyperekplexia and discontinued levetiracetam. Since the patient’s hyperekplexia symptoms were relatively mild, we did not administer clonazepam for treatment.

Outcome and follow-up: At 26 months of age (approximately 8 months postdiagnosis), we conducted a follow-up assessment of the patient. At this stage, the parents reported that the child’s convulsive seizures had completely resolved, with non-epileptic stimulus-induced symptoms also markedly reduced. The staring spells and limb rigidity episodes characteristic of hyperreflexia had decreased from occurring several times weekly to just 1 to 2 isolated incidents over the preceding 2 months. Notably, levetiracetam was discontinued following the diagnosis of hyperreflexia at 18 months of age, and clonazepam was not initiated at this stage for multiple reasons. Subsequently, the patient experienced no further clonic seizures. During the 8-month follow-up period (which included 2 episodes of acute upper respiratory tract infection), no epileptic seizures occurred. The child’s growth and developmental progress remained favorable.

## 3. Discussion

Hyperekplexia is a rare hereditary neurological disorder characterized by an exaggerated startle reflex and generalized rigidity in response to sudden stimuli^[4]^. More than 200 genetically confirmed cases have been reported to date^[5]^. The condition was first described in 1962 by Kok and Bruyn as“an unidentified hereditary disease.”^[6]^ Pathogenic variants in 4 major genes – GLRA1, SLC6A5, GPHN, and GLRB – have been implicated, with GLRA1 being the most frequent, accounting for 62.9% to 80% of cases.^[2,7]^

Schaefer et al identified a missense mutation (Q177K) in the extracellular β8-β9 loop of the GLRA1 subunit, which disrupts the hydrogen bonding network and impairs glycine receptor function by accelerating channel closure and reducing synaptic integration.^[8,9]^ In studies of the corresponding mouse model, mutant glycine receptors exhibited decreased current amplitudes, confirming a loss-of-function pathophysiology.

Typically, hyperekplexia is characterized by an exaggerated startle response, generalized rigidity, and a positive nose-tap test.^[10–12]^ Additional features may include infantile apnea, developmental delays, or abdominal hernias.^[12]^ Most patients have normal EEG and brain imaging findings.^[13]^ In contrast, our patient exhibited the characteristic stimulus-induced staring and limb stiffness, but notably demonstrated a negative nose-tap test and lacked other common comorbidities.

Our patient’s mild phenotype contrasts with the classic presentation in Chinese cohorts. Zhan et al (2020) reported 4 patients with homozygous GLRA1 mutations exhibiting neonatal hypertonia, positive nose-tap test, and recurrent falls requiring clonazepam.^[3]^ In comparison, our case with a heterozygous nonsense variant (p.Trp198Ter) demonstrated a negative nose-tap test, no significant falls, and milder symptoms not requiring medication, suggesting distinct pathogenesis between truncating and missense mutations.

Most patients experience symptomatic improvement with age.^[14]^ A notable exception is the case by Antonioni et al, where symptoms worsened over time and genetic testing was negative for known hyperekplexia-related genes.^[15]^ In contrast, Milenkovic et al and Kimura et al reported patients with confirmed GLRA1 mutations who responded excellently to clonazepam, a benzodiazepine that enhances GABAergic inhibition and can compensate for impaired glycinergic transmission.^[16]^

The initial diagnosis was challenging due to the patient’s atypical presentation. While she exhibited stimulus-induced staring and limb stiffness, her nose-tap test was negative, and she had no associated hernias or dysplasias. Her history of 3 febrile seizures further complicated the picture, leading to initial consideration of epilepsy syndromes such as Childhood Absence Epilepsy, Dravet Syndrome, or GEFS+. However, the self-limited nature of the episodes within a febrile context and normal EEG findings were more consistent with simple febrile seizures.

**Figure 2.**
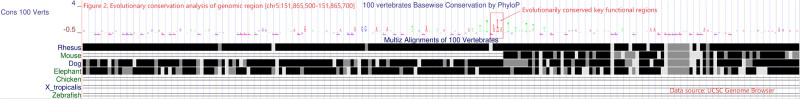
Evolutionary conservation of the genomic region encoding GLRA1 p.Trp198.(A) Multiz alignment of 100 vertebrate sequences demonstrates perfect conservation of the nucleotide (indicated by the arrow) corresponding to human GLRA1 c.593. (B) PhyloP basewise conservation scores show strong evolutionary constraint at this site. The high degree of conservation underscores the functional importance of this residue.

Whole-exome sequencing was pivotal in resolving this diagnostic dilemma, by identifying a de novo heterozygous nonsense variant in the GLRA1 gene (c.593G > A, p.Trp198Ter). This genetic finding, when integrated with her clinical features, confirmed the diagnosis of hyperekplexia. The p.Trp198Ter variant introduces a premature stop codon in the extracellular domain of GLRA1, truncating the protein before its transmembrane and intracellular regions. The affected tryptophan residue is highly conserved across species (Fig. 2), underscoring its functional importance. We hypothesize that the expression of a truncated extracellular domain may partially preserve receptor assembly or ligand interaction, potentially explaining the atypical negative nose-tap test and milder phenotype observed in our patient.

We propose that this specific truncating mutation – which likely produces a partially functional receptor fragment – directly explains the atypical clinical features: the preserved extracellular domain may account for the negative nose-tap test, while the overall glycineergic deficit likely lowered the seizure threshold, predisposing her to febrile provocation. This genetic finding confirmed the diagnosis of hyperekplexia and prompted the discontinuation of levetiracetam.

In addition, the nonsense variant (p.Trp198Ter) in our case suggests a distinct pathogenic mechanism from the commonly reported missense mutations in GLRA1. Typically, missense variants act through a dominant-negative effect, where the mutant subunit disrupts the entire receptor complex. In contrast, our truncating variant likely causes haploinsufficiency, resulting in a complete loss of the mutant allele’s function. This fundamental difference may explain the milder, atypical phenotype (e.g., negative nose-tap test) in our patient, as the absence of a functional protein may be less detrimental than the presence of a malicious one that poisons the glycine receptor complex.

According to previous reports in the literature, clonazepam was usually the hyperekplexia of treatment, but our children did not continue to take clonazepam after levetiracetam was discontinued. This is mainly based on the following reasons: First, the child has only had 3 convulsion seizures since birth, all accompanied by fever, and the symptoms are mild (no frequent falls, nocturnal myoclonic convulsions, etc); Second, clonazepam belongs to benzodiazepines, which enhances glycinergic inhibition by modulating GABA_A receptors, but is usually only used for patients with severe symptoms, and side effects (drowsiness, respiratory depression and addiction, etc). Therefore, after full discussion with the parents, we decided to continue clinical observation without immediate use of clonazepam.

## 4. Conclusion

We report the history of a case of hypersensitive startle response disorder caused by a novel nonsense mutation, c.593G > A (p.Trp198Ter), in the *GLRA1* gene, a locus that has not been reported in the literature. The clinical features of the children showed hypersensitivity to external sound and tactile stimuli, with occasional induced dullness of the eyes, stiffness of the limbs, and a negative nose-pointing test. Our study enriches the clinical phenotype of *GLRA1* mutations and provides clinical support for the management of hyperarousal syndrome.

## Acknowledgments

We thank the patient and her family members for their contributions to this study.

## Author contributions

**Writing** – **original draft:** Shuang-Zhu Lin, Xiao-Yu Sun, Yang-Yang Tan, Yang-Fan Qi, Kai Jiang.

**Writing** – **review & editing:** Shuang-Zhu Lin, Xiao-Yu Sun, Yang-Fan Qi, Kai Jiang.
